# MRI Application and Challenges of Hyperpolarized Carbon-13 Pyruvate in Translational and Clinical Cardiovascular Studies: A Literature Review

**DOI:** 10.3390/diagnostics14101035

**Published:** 2024-05-17

**Authors:** Francesca Frijia, Alessandra Flori, Giulio Giovannetti, Andrea Barison, Luca Menichetti, Maria Filomena Santarelli, Vincenzo Positano

**Affiliations:** 1 Bioengineering Unit, Fondazione Toscana G. Monasterio, 56124 Pisa, Italy; alessandra.flori@ftgm.it (A.F.); positano@ftgm.it (V.P.); 2 Institute of Clinical Physiology, National Research Council (CNR), 56124 Pisa, Italy; giulio.giovannetti@ifc.cnr.it (G.G.); luca.menichetti@ifc.cnr.it (L.M.); santarel@ifc.cnr.it (M.F.S.); 3 Cardiology and Cardiovascular Medicine Unit, Fondazione Toscana G. Monasterio, 56124 Pisa, Italy; andrea.barison@ftgm.it

**Keywords:** hyperpolarized magnetic resonance, dynamic nuclear polarization, carbon-13, pyruvate, cardiac metabolism

## Abstract

Cardiovascular disease shows, or may even be caused by, changes in metabolism. Hyperpolarized magnetic resonance spectroscopy and imaging is a technique that could assess the role of different aspects of metabolism in heart disease, allowing real-time metabolic flux assessment in vivo. In this review, we introduce the main hyperpolarization techniques. Then, we summarize the use of dedicated radiofrequency ^13^C coils, and report a state of the art of ^13^C data acquisition. Finally, this review provides an overview of the pre-clinical and clinical studies on cardiac metabolism in the healthy and diseased heart. We furthermore show what advances have been made to translate this technique into the clinic in the near future and what technical challenges still remain, such as exploring other metabolic substrates.

## 1. Background

Magnetic resonance imaging (MRI) and magnetic resonance spectroscopy (MRS) are powerful medical techniques able to provide detailed anatomical and functional clinical information in a non-invasive manner. MRI and MRS obtain structural and metabolic information noninvasively from nuclei spins that are naturally present in the human body, such as hydrogen nuclei in water and fat. Protons (^1^H) are the most commonly used nuclei due to their high gyromagnetic ratio and natural abundance in the human body and represent the basis of MRI. Other well-established nuclei commonly used in MRS are phosphorus (^31^P) [1], carbon (^13^C) [2], sodium (^23^Na) [3], and xenon (^129^Xe) [4]. In Table 1, we report the gyromagnetic ratios and the natural abundance of the mentioned nuclei in a magnetic field of 3 T [5]. 

Cardiac MRS allows in vivo detection and quantification of myocardial metabolism [6]. ^1^H spectroscopy enables the identification of creatine, triglycerides and lipids. ^31^P MRS represents the most widely used technique for myocardial bioenergetics studies. ^13^C MRS allows the identification of glucose, lactate, pyruvate, alanine, and bicarbonate, enabling quantification of myocardial metabolism and pyruvate dehydrogenase [7]. Hence, many clinical applications are focused on ^13^C spectroscopy. ^13^C spectra assure a large spectral range (162–185 ppm) and narrow line widths [8]. Unfortunately, ^13^C spectroscopy has a low sensitivity due to the low gyromagnetic ratio (a quarter with respect to ^1^H) and a low natural abundance in vivo (about 1%). Hence, techniques able to enhance the ^13^C signal are of great interest, as a higher signal intensity can provide improved sensitivity and contrast. Hyperpolarization techniques are able to enhance signal intensities by several orders of magnitude and thus largely overcome the major disadvantage of relatively low sensitivity [9]. Applications of hyperpolarization techniques include metabolic imaging, cancer detection, and studying molecular processes in real time [10]. Such in vivo studies have been carried out by researchers both in animals and, much more recently, in humans. In the latter case, cancer—especially brain cancer, but also prostate, kidney, and breast cancer—is the most studied pathology [11]. However, it is well recognized in the literature that the use of hyperpolarized ^13^C for cardiac studies in humans can provide very important metabolic information on the state of the heart, for example, on its viability during or after a heart attack [12]. Currently, hyperpolarized cardiac magnetic resonance imaging (CMR) can provide additional information that current myocardial viability assessments are unable to provide [13]. Indeed, CMR hyperpolarized ^13^C-pyruvate could potentially give additional information compared to conventional CMR and/or cardiac positron emission tomography (PET) due to the added metabolic measurements available [10,14]. The current results suggest that hyperpolarized CMR holds great potential, so much so that clinical adoption is starting to be proposed [15].

In this review, we first introduce the principal hyperpolarization techniques used, then show applications with dynamic nuclear polarization (DNP). We focus on applications in MR clinical scanners, reporting on dedicated radio frequency (RF) ^13^C coils and describing the MRI sequences used for ^13^C data acquisition. Furthermore, the review provides an overview of the pre-clinical studies on large animals and clinical studies on cardiac metabolism in healthy and diseased hearts.

## 2. Brief Overview of Hyperpolarization and Dissolution–Dynamic Nuclear Polarization

**Figure 1:**
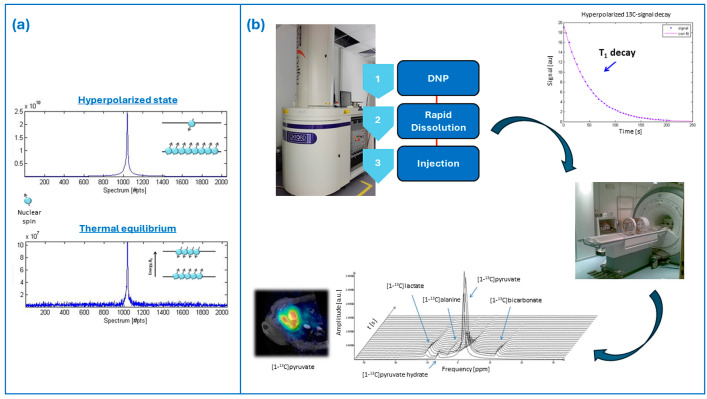
Panel (**a**) schematic representation of the hyperpolarized state; panel (**b**) hyperpolarization via d-DNP and schematic pipeline of the typical experiment with hyperpolarized contrast agents.

In the context of MRI and MRS studies, hyperpolarization refers to advanced techniques providing a major increase of the nuclear polarization and, therefore, of the signal-to-noise ratio (SNR). Hyperpolarization deals with the achievement of a so-called hyperpolarized state, in which the population difference between the nuclear energy states is increased by several orders of magnitude (Figure 1a). For spin ½ nuclei (such as ^1^H or ^13^C), this population difference, or polarization, is commonly defined as P = (N^+^ − N^−^)/(N^+^ + N^−^), where N^+^ and N^−^ are the populations of the two possible spin states. Since the MR signal is directly proportional to the polarization P, hyperpolarization will result in a conspicuous enhancement of the MR signal and the SNR [16]. The hyperpolarized state is a non-equilibrium condition of the nuclear spin system, which means that once the hyperpolarization process is concluded, the nuclear polarization recovers its thermal equilibrium through relaxation processes governed by the T_1_ relaxation time constant, leading to the decay of the hyperpolarized MR signal. Several hyperpolarization techniques were proposed in the literature. We only briefly mention here the most common hyperpolarization techniques that, to date, include the brute force method, the spin exchange by optical pumping (SEOP) method, the parahydrogen-induced polarization (PHIP) method, and the dynamic nuclear polarization (DNP) method. Moreover, long-lived spin states (LLSs) allow the investigation of various slow processes and sustain spin hyperpolarization, providing large NMR signal enhancements [17]. Relaxation times reach tens of minutes or even more for ^15^N and ^13^C spins [18,19]. 

The brute force method represents the most straightforward hyperpolarization approach based on only a strong magnetic field and low temperature. Operatively, the sample to be polarized is kept at high field (14 T) and low temperature (∼100 mK < T < 4 K) until the establishment of a thermal equilibrium polarization [20]. The brute force method does not use polarizing agents (or other modifications) or microwave irradiation. The technique was first exploited for gas hyperpolarization [21,22] and later for liquid-state tracers. In this latter case, the frozen sample is ejected from the polarizer after hyperpolarization and rapidly dissolved with hot H_2_O before imaging. Given the slow solid-state spin relaxation, transport of the hyperpolarized sample has also been demonstrated [23]. However, the achieved polarization levels are critical for in vivo applications. 

The first hyperpolarization technique applied in human studies is the so-called SEOP, leading to the production of hyperpolarized noble gases, such as ^3^He and ^129^Xe. In SEOP, circularly polarized light is exploited to optically pump rubidium (Rb) electrons and generate a highly spin-polarized Rb gas; the spin polarization is then transferred to noble gas nuclei through spin-exchange collisions. ^129^Xe proved to be a suitable gas for clinical studies where it can be used for functional MRI in the lungs [24,25,26] among other applications [27,28]. Techniques such as parahydrogen-induced polarization (PHIP) and dynamic nuclear polarization (DNP) showed great promise for the production of injectable hyperpolarized contrast agents in the liquid state.

The PHIP and DNP methods carry on the ex situ hyperpolarization of small isotopically enriched molecules, typically metabolic substrates, which can be subsequently administered in vivo, mainly for metabolic studies. ^13^C is, to date, the most used isotope due to some favorable characteristics, such as its lack of background signal in vivo, a T1 suitable for in vivo studies, and its sparse spectra. In PHIP, hyperpolarized compounds are produced through a chemical reaction, allowing the transfer of spin polarization from parahydrogen to a target molecule. The technique provides faster hyperpolarization and is technologically less demanding than DNP, with minor operational and maintenance costs; however, the yield in terms of final achieved polarization is generally lower. The first applications of PHIP were restricted to just a few molecules, essentially precursors ensuring direct hydrogenation for polarization transfer. More recent technological advancements—in particular, the parahydrogen-induced polarization-side arm hydrogenation (PHIP-SAH) [29,30] and the signal amplification by reversible exchange (SABRE) [31] methods—extended the applicability of the technique to several probes and led to reliable hyperpolarization and in vivo application of pyruvate; accordingly, PHIP is attracting increasing attention from the scientific community [32]. In the DNP technique, the high polarization of electron spins is used to enhance the nuclear spins polarization [33]. 

Human and translational studies on large animal experimental models with hyperpolarized tracers, which are the focus of this review, followed the development of the instrumentation for the so-called dissolution DNP (d-DNP) [9,34,35]. From an operational point of view, d-DNP in this kind of study is usually performed at a high magnetic field (3.35 to 7 T) and extremely low temperature (1.2–1.4 K). The liquid-state sample formulation is doped with a free radical (i.e., the source of polarization) and placed in a strong magnetic field and low temperature, where it forms a glass. Under such conditions, the unpaired electrons on the radical molecules are almost fully polarized and sample irradiation with low-power microwave mediates the transfer of such polarization to coupled nuclear spins. At the end of the polarization build-up, the frozen hyperpolarized sample is rapidly dissolved through rapid contact with a pressurized and heated dissolution medium to produce a hyperpolarized solution injectable in vivo (Figure 1b). Providing a comprehensive description of the different aspects regarding d-DNP, including radical and hardware development, is out of the scope of this review. We only mention here that upon the operating conditions of d-DNP, a homogeneous radical distribution is necessary for the efficient build-up of polarization and can be achieved by adding a biocompatible glassing agent, such as glycerol or DMSO, to the sample preparation [36,37,38]; moreover, the addition of small amounts of lanthanides such as Gd3+ can further improve the achievable polarization [39].

An interplay between different mechanisms considering the interaction between the electron and nuclear spins can be invoked to explain DNP. The contribution of these mechanisms to the hyperpolarization process depends on several factors, and in particular, the EPR linewidths of the polarizing agent (the radical) with respect to the nuclear Larmor frequency. However, a detailed explanation of the theoretical aspects of DNP is not the purpose of this review and we refer to the specialized literature for further discussion [40,41,42,43,44,45]. An increase in the signal-to-noise ratio by four orders of magnitude has been reported for 13C-labeled compounds using d-DNP [9], but the yield in terms of achieved polarization is dependent on the hyperpolarized molecule and nuclear spins. In fact, d-DNP is a versatile and reliable hyperpolarization method: a few other nuclei have been explored besides ^13^C, including ^15^N [46], ^29^Si [47] and ^1^H [48]. The development of a d-DNP hyperpolarizer working in a sterile environment paved the way for direct translation of the technique to the clinics [49]. The d-DNP method has been recently approved for human studies, and several clinical trials have been approved and are ongoing to date, most of them focusing on cancer [50,51].

More recent advancements in d-DNP address some of the limitations of the technique to further increase the available signal-to-noise ratio and the lifetime of the hyperpolarization [52]. Novel approaches in sample formulation have been reported—for instance, avoiding the presence of the glassing agent and increasing the nuclear spin concentration [53] or using radicals incorporated into a mesostructured silica material (HYPSOs) [54] or UV-induced nonpersistent radicals [55], which are then removed from the hyperpolarized solution before in vivo injection. Among the most relevant instrumentation improvements, the development of a cryogen-free d-DNP polarizer [56] holds promise for reducing the maintenance and operational costs of the original d-DNP configuration.

Unlike conventional MR contrast agents based on gadolinium, hyperpolarized tracers directly produce the detected MR signal; therefore, the signal intensity in hyperpolarized MR studies is proportional to the contrast agent concentration in tissue and the MR acquisition is characterized by the lack of background signal. As previously mentioned, the hyperpolarized MR signal decays over time through relaxation processes governed by the T_1_ relaxation time constant. T_1_ is on the order of units of tens of seconds at clinical magnetic fields for the isotopically enriched molecules typically used within in vivo studies (the nominal T_1_ for the gold standard ^13^C-pyruvate being 60 s). Furthermore, excitation through RF pulses during the MR acquisition irreversibly contributes to destroying the gained polarization. The rapid decay of the hyperpolarization, and consequently of the detectable MR signal, has two important implications: first, only fast metabolic processes with a timescale of a few minutes can be investigated using this approach; second, a specific MR acquisition setup, dealing with both sequences and RF coil design, should be developed to provide fast acquisition of the hyperpolarized signal with high spectral and spatial resolution [57,58]. This is especially challenging when dealing with a moving organ such as the heart.

## 3. Biological and Technical Considerations of Pyruvate Metabolism

**Figure 2:**
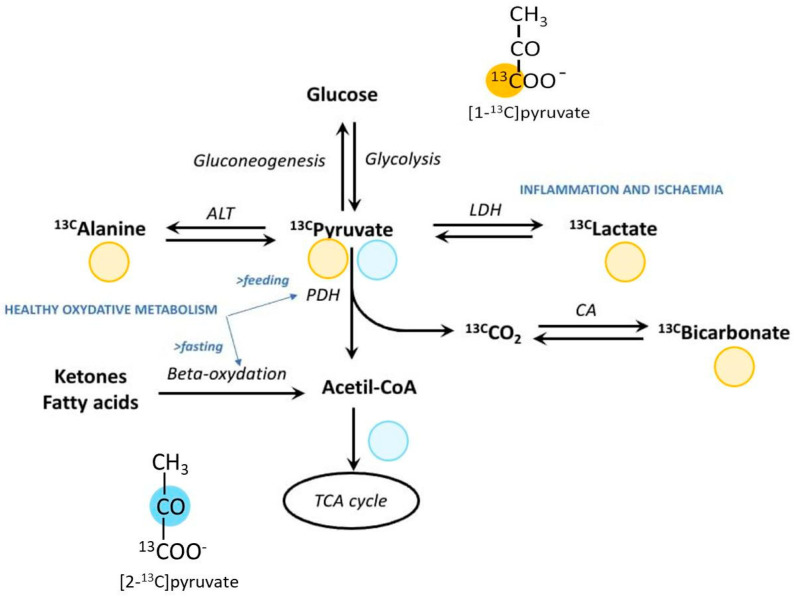
In the heart, ^13^C-pyruvate may undergo transamination to ^13^C-alanine, reduction to ^13^C-lactate or oxidation to ^13^C-CO_2_ and acetyl-CoA (Ac-CoA). ^13^C-CO_2_ is rapidly converted to ^13^C-bicarbonate by carbonic anhydrase. Acetyl-CoA is metabolized in the tricarboxylic acid (TCA) cycle. *Please note that the study of Acetyl-CoA and TCA metabolites requires labeling of pyruvate in C2 position ([2-^13^C]pyruvate). ALT, alanine transaminase; CA, carbonic anhydrase; LDH, lactate dehydrogenase; PDH, pyruvate dehydrogenase.

To date, pyruvate represents the leading probe for hyperpolarization in vivo studies and is, as far as we know, the only molecule that has received approval for human studies. This is essentially due to a twofold reason: on the one hand, pyruvate provides the best chemical and physical properties for d-DNP, including a high ^13^C concentration and a long T_1_ relaxation time (around 60 s); moreover, pyruvic acid is a self-glassing compound, which means that it is not necessary to add other glassing agents to the DNP formulation. On the other hand, pyruvate is an endogenous metabolic substrate which undergoes a rapid metabolic conversion once injected in vivo, in a time frame compatible with the hyperpolarization decay, thus allowing the real-time observation of its main metabolic products through sensitivity-enhanced MRS, namely lactate and bicarbonate. Pyruvate is an intermediate of the cell glucose metabolism at the crossroad between glycolysis and oxidative phosphorylation (Figure 2).

Pyruvate is transported across the cell membrane by the family of the monocarboxylate transporters (MCTs). Under anaerobic conditions in the cytosol pyruvate is converted into lactate in a reaction catalyzed by the lactate dehydrogenase (LDH) enzyme and into alanine by the reaction catalyzed by the enzyme alanine transaminase (ALT). Under aerobic conditions, pyruvate enters the mitochondria, where the pyruvate dehydrogenase complex (PDH) regulates the conversion into Acetyl-CoA, which takes part in the Krebs (or TCA) cycle, where it is further oxidized. In parallel, inside mitochondria, pyruvate is also converted into CO_2_, which rapidly equilibrates with bicarbonate through the action of the enzyme carbonic anhydrase (CA). The hyperpolarization of pyruvate and the detection of its downstream metabolites provides a unique opportunity to track and investigate the balance between glycolysis and oxidative metabolism in different physio-pathologic conditions, in a non-invasive manner and in real time. In fact, hyperpolarization of the ^13^C label of the carboxylic group (C1 position) allows tracking of the glycolytic pathway, while the hyperpolarization of pyruvate labeled in the C2 position (on the carbonyl group) allows exploring the oxidative and TCA cycle metabolism.

In healthy tissue, the major energy expenditure of the cell is supplied by oxidative phosphorylation in mitochondria; however, in hypoxia or in certain pathologic conditions, an increase in energy production through glycolysis can be observed. For instance, cancer cells are characterized by a different glucose metabolism to provide the energy required to support their rapid proliferation: due to the so-called Warburg effect, in cancer cells, the mitochondrial oxidative phosphorylation is replaced with cytosolic glycolysis even in the presence of oxygen (aerobic glycolysis). Detecting this metabolic alteration in tumor tissue, which results in increased conversion of pyruvate into lactate, is currently the main purpose of most of the d-DNP studies in pre-clinical models and in humans [11].

The healthy adult heart relies mainly on the β-oxidation of free fatty acids in the myocardium (60%-90%) to produce the energy necessary to support the contractile activity, while the remaining energy derives from the oxidation of pyruvate, ketone bodies and amino acids [59,60]. Inside cardiomyocytes, fatty acids enter the mitochondrial matrix and are oxidized by the carnitines palmitoyltransferase type 1 and type 2 (CPT-1 and CPT-2), and the carnitine acylcarnitine translocase (CT) enzymes [61]. The balance between glucose and fatty acids oxidation for ATP (and hence energy) production is highly regulated and depends on several factors; in particular, the PDH complex is a key enzyme and represents an interesting target for investigating substrate selection in cardiomyocytes.

A metabolic shift towards increased glucose oxidation has been observed in cardiac diseases such as heart failure and ischemia [62]; conversely, a shift towards fatty acids oxidation has been observed in diabetes. Hyperpolarized pyruvate in cardiac studies provides the opportunity to assess the flux of the PDH and LDH enzymes through the detection of bicarbonate and lactate, respectively, as well as to investigate the balance between aerobic and anaerobic metabolism in the myocardium, in different physio-pathological conditions. Because fatty acids are the main energy fuel of the myocardium, they represent an interesting hyperpolarizable probe for cardiac metabolic studies. Short and medium-chain fatty acids are relatively small molecules and can be successfully hyperpolarized with d-DNP. Accordingly, ^13^C-labeled short and medium-chain fatty acids such as acetate [63,64], butyrate [65,66] or octanoate [62] have been proposed as alternative hyperpolarized probes to provide complementary information on cardiac metabolism in pre-clinical experimental models.

Finally, injection of hyperpolarized ^13^C-urea, a small molecule with favorable d-DNP properties that is not metabolized in the time span of the typical hyperpolarized MR experiment, provides the opportunity to investigate cardiac perfusion in real-time [67]. In particular, the co-polarization of urea and pyruvate can, in principle, provide the investigation of myocardial perfusion and metabolism non-invasively and in a single study [68].

## 4. ^13^C Radiofrequency Coils

In MR experiments, the RF field is generated by a transmit coil and picked up by a receive coil [69]. Since the extension of the region to analyze is not known a priori, the transmit coil must produce a highly homogeneous magnetic field in the desired field of view (FOV). To achieve this, transmit coils are usually large in order to optimize the magnetic field homogeneity in a significant tissue volume. Conversely, the receive coil must maximize signal detection while minimizing noise, and for this purpose, its size must be minimized. When choosing a coil setup for an MR experiment, both transmit and receive RF coils must be adapted to the specific application and to the human/animal body portion dimension and shape, although they have to provide good performances with slightly different subjects. According to their shapes, coils can be categorized into volume, surface, and phased-array coils [70]. Volume coils are often employed both as transmit and receive coils since they can generate a homogeneous magnetic field in a large region surrounding the sample portion. Surface coils are much smaller than volume coils because they must guarantee high SNR in the images, even if with relatively poor magnetic field homogeneity [71]. Finally, phased-array coils [72] allow the achievement of good SNR images, typical of surface coils, with a large sensitivity region, usually obtained with volume coils. They are also employed in parallel imaging applications [73], where the magnetic field spatial variation of the single coil elements permits the signal spatial encoding, which provides substantial reductions in image acquisition time. However, the ideal coil setup should comprise the use of two different coil configurations: a transmitter highly homogeneous volume coil and a receiver high local sensitivity (surface or phased-array) coil [74]. For ^1^H/^13^C MR imaging and spectroscopy experiments conducted on clinical MRI scanners, the system must be equipped with multinuclear spectroscopy capability [75] for operating at two different frequencies and by using two different coil setups. The proton images, necessary for providing an anatomical reference for registration [51], are acquired by using the built-in whole-body coil or a cardiac array receiver coil. For the carbon image acquisition, dedicated coils placed inside the bore must be employed [76]. In particular, transmit ^13^C coils are mainly constituted by Helmholtz and birdcage coils, while receive ^13^C coils by surface and phased-array coils [51,74]. However, in some applications, a custom-built transmit/receive ^13^C surface coil was employed, both in single [77,78,79] or multi-element [67] configurations, especially when a specific heart portion must be interrogated. A multiple-channel surface coil provides a high SNR close to the coil surface but a non-uniform sensitivity in depth, while volumetric coils provide a uniform sensitivity over the FOV, at the cost of lower SNR. An example of the performance of a flexible 16-channel phased array coil for application in pig heart studies was reported, assessing both SNR and signal uniformity in phantom and animal experiments [80].

Clearly, the ideal hardware setup would require the use of a single transmit/receive dual-tuned coil operating at ^1^H and ^13^C nucleus frequencies by guaranteeing data acquisition in sequence without disturbing and repositioning the patient [81,82]. An optimal coil design should guarantee the minimization of the interactions between the carbon and the proton signals, starting from the channels’ geometrical decoupling [70]. Moreover, for optimizing MR experiments, quadrature ^13^C coils producing/receiving circular polarized magnetic fields must be designed to reduce by a factor of two the power requirement in transmission and increase by a factor of √2 the received signal SNR [83,84]. Having ^13^C-nucleus a lower gyromagnetic ratio than that of ^1^H, the ^13^C coil tuning frequency (32.1 MHz at 3 T) is similarly reduced compared to ^1^H coil tuning frequency (128 MHz at 3 T). It means that if for ^1^H the sample noise dominates, ^13^C frequency has relatively more noise contribution from the coil with respect to sample [85], meaning that coil design has a bigger impact [57], although sample noise still is likely the dominant contributor for human/pig-sized coils [51]. However, the purchase of such ^13^C coils can be prohibitive for most centers [10]; therefore, homemade coil-building can be a cheaper solution for performing hyperpolarized MR experiments. 

To achieve this goal, the simulation and the design of RF coils can be carried out using two different methods. The first one is based on magnetostatic theory and implies the assumption of a nearly static field. Therefore, it is useful for the design of low-frequency-tuned coils, whose size is much lower than the wavelength. When the coil tuning frequency increases, the interaction between RF fields and the body becomes strong and full-wave methods based on Maxwell’s equation solutions have to be used, including the finite-difference time domain (FDTD) method, the finite element methods (FEM), and the method of moments (MoM). However, the computation times of such full-wave methods are much longer with respect to the magnetostatic approach [86,87].

**Figure 3:**
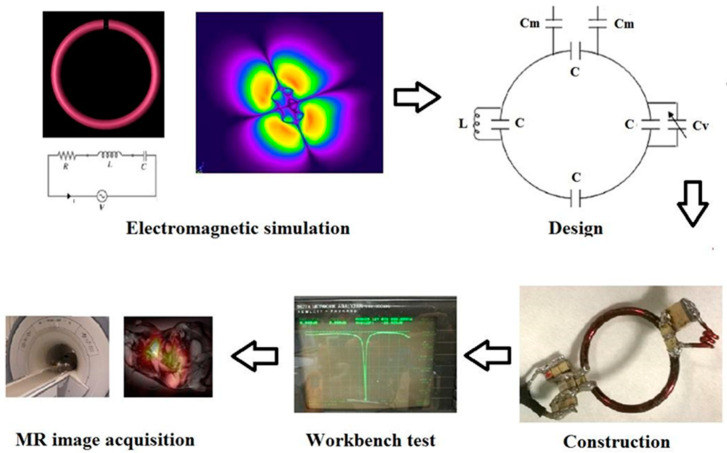
The different phases in the RF coil development.

Figure 3 summarizes the different phases necessary for RF coil construction in general, divided into simulation, design, and tests.

**Figure 4:**
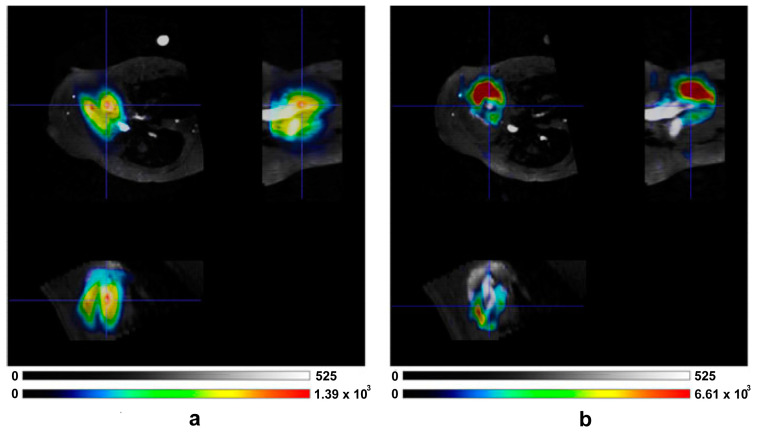
Hyperpolarized ^13^C-pyruvate and bicarbonate maps evaluated with the hybrid coil: (**a**) pyruvate signal, (**b**) bicarbonate signal. Reprinted by permission from Giovannetti et al., App Magn Reson 2013 [91].

As an example, simulation and design of homemade different ^13^C coil configurations for hyperpolarized studies on pig hearts with a clinical 3 T MRI scanner was carried out in our cardiovascular laboratory [88]. In particular, we initially designed a transmit/receive circular coil [89] which was compared with a commercial transmit/receive birdcage coil (Rapid Biomedical, Wurzburg, Germany) [90]. We then modified both coils to use the volume coil for transmission and the surface coil for reception (Figure 4) [91].

Another improvement in the SNR of the acquired signals was obtained by designing a quadrature surface coil configuration constituted by a circular and a butterfly coil employed in transmit/receive configuration [92] and in receive mode in combination with a birdcage coil as a transmitter [93].

## 5. ^13^C-MRI Image Acquisition

For ^13^C hyperpolarized acquisition, in addition to the use of dedicated RF coils, it is recommended to use a high-field scanner, as the spectral separation of pyruvate and its metabolites is greater, even though the larger field strength shortens the relaxation time of ^13^C-pyruvate [81]. Therefore, for a good-quality image acquisition, a compromise on the choice of the scanner must be reached; in the majority of published human ^13^C-pyruvate studies, a 3 T scanner has been used. A field strength of 3 T is well-suited also for ^1^H MRI anatomical reference and correlative imaging [51].

Hyperpolarized MRSI studies require specialized sequences due to the fast metabolism and the rapid decay of ^13^C. Various pulse sequences with different trade-offs between temporal and spatial resolution have been developed for measuring hyperpolarized nuclei, considering beyond those already mentioned the constraint that the radiofrequency induced signal further reduces the magnetization upon each excitation. The sequences used most often are based on a simple pulse-and-acquire frame in which a slice-selective pulse is combined with a specific gradient readout to encode the spectral and spatial dimensions [94].

The first approach for the study of metabolic imaging using hyperpolarized ^13^C-pyruvate consisted of acquiring 1D and 2D dynamic MRS at a temporal resolution (about 3 s) using ^13^C spectroscopic sequences (FIDCSI); with this sequence it is possible to acquire single spectra during one-minute acquisition after pyruvate injection [95] and follows the pyruvate signal and its metabolic derivatives signal: lactate, bicarbonate and alanine. At a first step in the ^13^C acquisition, transmit gain calibration is performed to adjust the RF power levels to the desired flip angles. Calibration is implemented on the basis of the Bloch–Siegert method [96] with a ^13^C-pyruvate phantom positioned in the coil sensitivity area and close to the imaging plane. From the same acquisition, the central frequency, line broadening, and SNR can also be determined.

**Figure 5:**
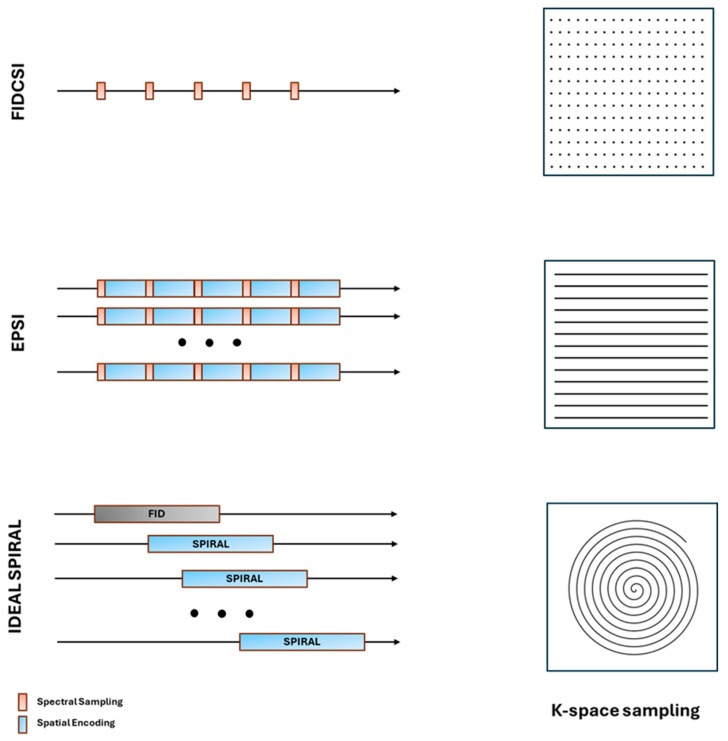
Schematization of the FIDCSI, EPSI, and IDEAL SPIRAL acquisition sequences and corresponding k-space sampling strategies.

Subsequently, the sequence used is echo-planar spectroscopic imaging (EPSI) for which, after one RF excitation, a single k-space is acquired and repeated in time; accordingly, the spectral and spatial dimensions are sampled simultaneously [94]. EPSI provides a significantly increased encoding efficiency relative to FIDCSI because it acquires a full line of k-space after each excitation [57]. The acquisition time for the biochemical pathways can be sampled dynamically with a time resolution of a few seconds. The EPSI sequence has a high gradient demand, and the spectral and spatial resolutions are limited by the maximum available gradient amplitude and slew rate. To avoid the need for high gradient hardware performances, Weisenger et al. [97] presented an efficient CSI scheme for hyperpolarized ^13^C metabolic imaging based on IDEAL single-shot spiral image encoding and echo-time shifting in between excitations for the CS encoding. To allow the mapping of the full spectrum rather than a limited number of peaks at certain prescribed frequencies, a free-induction decay (FID) spectrum is also acquired, and the obtained chemical shift prior knowledge was useful for the reconstruction. Figure 5 depicts the three main sequence schemes (FIDCSI, EPSI, and IDEAL SPIRAL) and the related k-space sampling strategies. 

**Figure 6:**
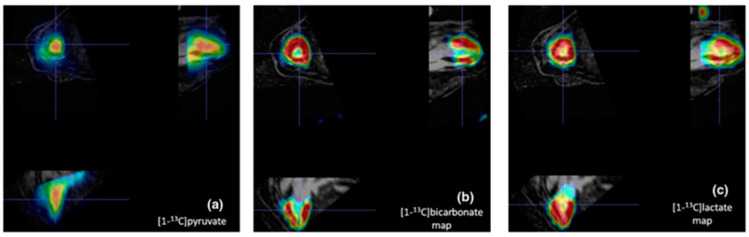
Hyperpolarized ^13^C-pyruvate (**a**), bicarbonate (**b**), and lactate maps (**c**) acquired by a 3D-IDEAL spiral CSI sequence. Reprinted by permission from Flori et al., App Magn Reson 2012 [98].

Figure 6 shows in vivo pig data acquired using a 3D-IDEAL spiral CSI sequence showing the spatial distribution of metabolites in the principal cardiac axes’ views of the heart.

To increase the spectral width that is crucial for acquiring all metabolite behavior, Durst et al. [94] describe a spiral chemical shift imaging (SPCSI) sequence that obtains spectral and spatial information simultaneously during readout by repeatedly scanning a spiral trajectory after RF excitation. In this case, the duration of a single spiral determines the spectral width. The SPCSI sequence was designed for two different regimes of spatial resolution: additional excitations with a time-shifted trajectory or the k-space sampling being split into multiple spiral interleaves. This approach permits having two-dimensional spatial and spectral information from a single excitation. Rapid multi-slice MR pulse sequence with the k-space trajectories is the most common acquisition approach for most cardiac studies [79]. For the ^13^C heart acquisition, the total length of the pulse is an important design parameter because it determines the width of the transition between frequencies that are excited versus those that are not perturbed by the RF pulse at all. This creates a design tradeoff between longer pulses with sharper transitions versus shorter pulses that enable a shorter sequence repetition time [79].

## 6. Clinical Applications from Pre-Clinical to Human Studies

Several pre-clinical and clinical studies with hyperpolarized ^13^C molecules have been performed in recent years. Many ^13^C-labeled agents have been successfully used in animal studies, including ^13^C-pyruvate and ^13^C-urea to assess perfusion, ^13^C-fumarate to detect necrosis, ^13^C-alpha ketoglutarate to assess isocitrate dehydrogenase (IDH) activity, ^13^C-butyrate as a measure of fatty acid metabolism, ^13^C-bicarbonate to assess extracellular pH, and ^13^C-dehydroascorbate to measure redox potential. ^13^C-acetate has been employed to assess TCA flux and fatty acid oxidation in heart and skeletal muscles through its conversion to acetyl-CoA by acetyl-CoA synthase. ^13^C-glucose has been used to monitor flux via the pentose phosphate pathway as well as glycolytic flux and lactate production. ^13^C-alanine has been employed as an alternate probe to study metabolism in the muscle and liver. Several other biologically relevant, potentially polarizable molecules remain to be studied [99]. Table 2 lists the most common ^13^C probes used in pre-clinical and clinical studies.

Most pre-clinical and clinical studies with hyperpolarized ^13^C compounds are oncologic [100]. The first human study investigating hyperpolarized ^13^C-pyruvate MRI in cancer was published in 2013: high prostatic ^13^C-lactate signal was demonstrated in a patient who had no abnormal signal intensity on conventional proton MR images, suggesting that clinical hyperpolarized ^13^C-pyruvate MRI could depict some tumors earlier than conventional ^1^H MR images [101]. Since then, the metabolism of hyperpolarized ^13^C-pyruvate to hyperpolarized ^13^C-lactate has been demonstrated in several tumors, including those of the prostate, pancreas, kidney, breast, and brain. Hyperpolarized ^13^C MRI in oncology is virtually able to stratify tumors by grade, select therapeutic pathways based on tumor metabolic profiles, and detect early treatment response through the imaging of the metabolism shifts that precede tumor structural changes [11,102]. In gliomas, as an example, it has been demonstrated that hyperpolarized ^13^C-pyruvate can detect metabolic subtypes, which can be dichotomized into more glycolytic and oxidative subtypes that have differing drug and radiation sensitivities [103,104].

Apart from tumors, pyruvate metabolism has also been studied in the neurological field, including traumatic brain injury and neurodegenerative diseases: measurements of hyperpolarized ^13^C pyruvate metabolism in the human brain can be used to measure regional variations in metabolism in physiological and pathological conditions [105]. While hyperpolarized ^13^C-pyruvate has been successfully utilized as a probe to quantify the conversion to ^13^C-lactate and ^13^C-bicarbonate in the human brain, its metabolism and conversion to ^13^C-CO_2_ through PDH prevents direct detection of TCA cycle metabolism. For this reason, hyperpolarization of ^13^C-pyruvate in the C2 position has been performed to provide a unique MR molecular imaging window into the TCA cycle as the labeled carbon is carried over to acetyl-CoA and enables the observation of [5–^13^C]glutamate after enzyme-catalyzed conversion from ⍺-ketoglutarate [106]. More recently, a novel hyperpolarized ^13^C MR RF pulse sequence has been applied in five healthy volunteers for acquiring volumetric and dynamic EPI of hyperpolarized [2-^13^C]-pyruvate metabolism to [5-^13^C]-glutamate and to [2-^13^C]-lactate, i.e., probing oxidative and glycolytic simultaneously [107]. In the human brain, glutamate is the most abundant free amino acid and is at a crossroad between multiple metabolic pathways, and hyperpolarized MR imaging holds promise to unveil new pathophysiological insights into neurological disorders. In kidney diseases, hyperpolarized ^13^C pyruvate allows to assess the underlying metabolic and pathophysiological changes [108]. The greater sensitivity and specificity of hyperpolarized [1-^13^C]pyruvate can be used as an early marker of disease progression and treatment response in clinical trials [109].

### 6.1. Pre-Clinical Cardiovascular Studies in Large Animal Models

Hyperpolarized ^13^C CMR has been used in a variety of pig models in the context of heart failure, including right ventricular heart failure [110], dilated cardiac myopathy [78], and ischemia/reperfusion injury [83,111] (Table 3). In heart failure, there is increased glucose metabolism through enhanced glycolysis, but at the same time, oxidative phosphorylation is depressed due to impaired flux through PDH. Similarly, in myocardial ischemia, compromised coronary blood flow and subsequent lack of oxygen supply drive a metabolic switch to increased anaerobic glycolysis and hence lactate production [81]. For these reasons, a relative increase in relative lactate-to-bicarbonate appears to be a common marker in heart disease, albeit with different dynamics over disease progression. Infusion of hyperpolarized ^13^C pyruvate before and immediately after ischemia has also been used to monitor intracellular pH through the HCO_3_/CO_2_ ratio using the Henderson–Hasselbalch equation and resulted in good agreement with ^31^P MRS measurements of pH [112]. In a study on ischemia/reperfusion injury (performed with a pneumatic occluder placed around the left anterior descending artery in seven pigs), hyperpolarized ^13^C-pyruvate imaging was performed at rest, during coronary occlusion, and 5 min after reperfusion. During occlusion, a decrease in ^13^C-lactate and ^13^C-bicarbonate was found in myocardial ischemic segments compared to remote segments. During reperfusion, the ^13^C-lactate signal increased in ischemic segments, while ^13^C-bicarbonate was persistently reduced [113]. An experimental study on eight pigs (five with catheter-induced myocardial infarctions, three controls) undergoing serial hyperpolarized ^13^C MR imaging (before infarction and at 6 days, 5 weeks, and 9 weeks postinfarction) revealed temporarily elevated lactate-to-bicarbonate ratios at Day 6 in the infarcted relative to remote myocardium [14]. The temporal changes of lactate-to-bicarbonate ratios were found to correlate with changes in T2 and impaired local contractility. Although LDH is known as a key factor in anaerobic cellular respiration under ischemic conditions, increased ^13^C-lactate production has also been associated with the monocyte/macrophage inflammatory response. Assessment of PDH flux via the hyperpolarized ^13^C bicarbonate signal revealed recovery of aerobic cellular respiration in the hibernating myocardium, which correlated with recovery of local radial strain. 

Besides metabolic imaging, ^13^C can also be used for angiographic applications, because ^13^C hyperpolarized contrast media can be imaged at a resonance frequency other than that of protons. The intracoronary injection of hyperpolarized ^13^C-hydroxyethylproponate has been tested in five pigs. With projection imaging using a fully balanced SSFP pulse sequence, angiograms of the right and left coronaries of the beating heart were obtained, with a SNR value in the range of 10–40 [84]. More recently, first-pass myocardial perfusion imaging using hyperpolarized ^13^C-urea has been performed in six pigs, with good data quality compared to conventional Gd-based contrast agents [67].

### 6.2. Human Cardiovascular Studies

Several human studies can be found in the literature (Table 4). In 2016, the first hyperpolarized ^13^C metabolic magnetic resonance imaging (MRI) of the human heart was reported in four healthy subjects [74]. After injection of 0.1 mmol/kg hyperpolarized ^13^C-pyruvate, its signal appeared within the chambers but not within the muscle. Imaging of the downstream metabolites showed the ^13^C-bicarbonate signal mainly confined to the left ventricular myocardium whereas the ^13^C-lactate signal appeared both within the chambers and in the myocardium. A recent report on two patients with acute myocardial infarctions undergoing hyperpolarized ^13^C-pyruvate imaging showed that nonviable segments with transmural infarction show reduced PDH-mediated aerobic conversion to ^13^C-bicarbonate, while viable segments have preserved ^13^C-bicarbonate signal. Similarly, ^13^C-lactate signals were absent in nonviable segments but were seen in viable segments [12].

**Figure 7:**
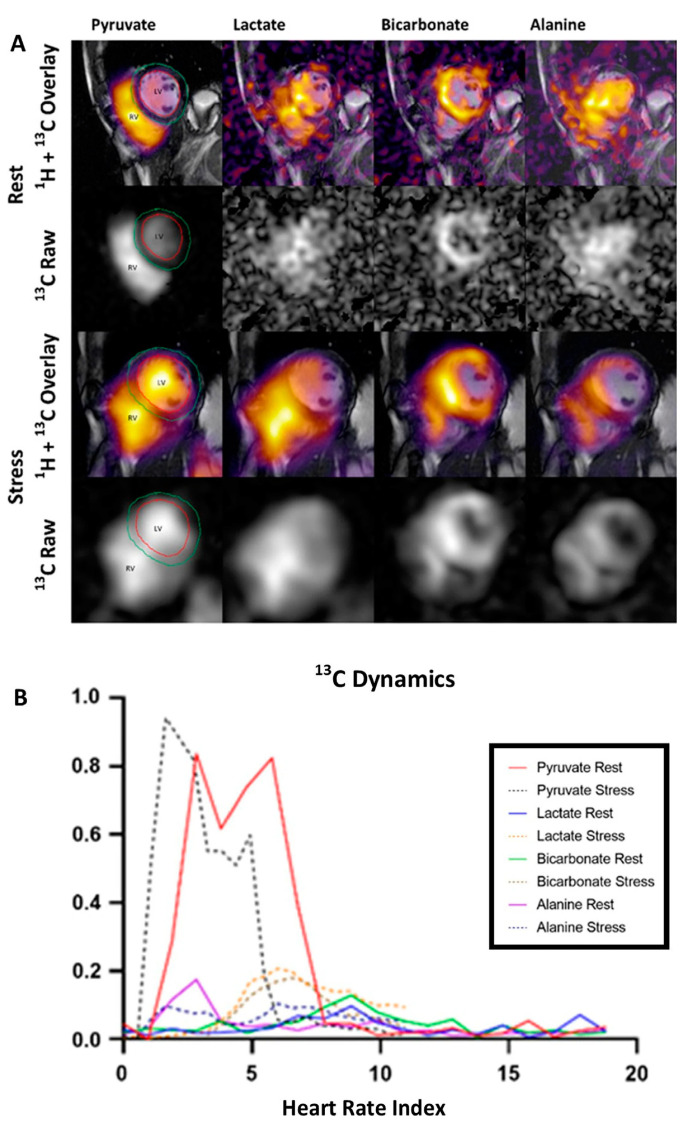
(**A**): Metabolic maps of ^13^C-pyruvate, ^13^C-lactate, ^13^C-bicarbonate, and ^13^C-alanine map. Metabolite data are shown overlaid with an anatomical cine image and as raw metabolite images. (**B**): Temporal dynamics for ^13^C-pyruvate and dynamics for ^13^C-pyruvate and its downstream metabolites from arrival of pyruvate in the lumen of the right ventricle (RV) and left ventricle (LV) to downstream appearance of ^13^C-lactate, ^13^C-bicarbonate, and ^13^C-alanine. Reprinted with permission from Joergensen et al., J Cardiovasc Magn Reson, 2022 [76].

In a study on 5 diabetic patients, cardiac metabolic flux through cardiac PDH (assessed by the ^13^C-bicarbonate to ^13^C-pyruvate ratio) was significantly reduced compared to 5 healthy controls, while the lactate dehydrogenase pathway (reflected by the ^13^C-lactate to ^13^C-pyruvate ratio) was increased. Transamination of ^13^C-pyruvate to ^13^C-alanine, which is proportional to the intracellular availability of pyruvate, was not different between patients and controls. After a 75 g oral glucose challenge, a significant increase in metabolic flux through PDH was observed (reflected by an increased ^13^C-bicarbonate to ^13^C-lactate ratio) [15]. In a study on six healthy subjects undergoing cine CMR and HP ^13^C-pyruvate CMR at rest and during adenosine stress, myocardial ^13^C-pyruvate perfusion was significantly increased during stress, accompanied by an overall increase of both ^13^C-lactate and ^13^C-bicarbonate. Adenosine stress testing combined with HP ^13^C-pyruvate CMR was not only feasible and well-tolerated but also successful in demonstrating an increased pyruvate oxidation during cardiac stress [76] (Figure 7).

In particular, the increased ^13^C-pyruvate signal was explained by an increased myocardial uptake and/or an increased vascular signal due to coronary vasodilation. The increase in ^13^C-lactate signal was explained by ^13^C-pyruvate to ^13^C-lactate exchange (which depends on pyruvate concentration), but the increase in PDH flux was even larger, demonstrating that the healthy heart increases oxidative energy production during moderate stress. The feasibility of using dual-labeled hyperpolarized [1,2-^13^C]-pyruvate as a substrate for dynamic cardiac metabolic studies was demonstrated in phantoms and in pigs [77]. [2-^13^C]-pyruvic acid has been also administered in three healthy subjects under both fasting and oral glucose load conditions; key downstream metabolites of [2-^13^C]-pyruvate metabolism in the heart included glycolytic derivative [2-^13^C]-lactate, TCA-associated metabolite [5-^13^C]-glutamate, and [1-^13^C]-acetylcarnitine, all of which increased after glucose load [75].

Currently, there are some ongoing clinical studies based on ^13^C-hyperpolarization, which are focusing on cardiac metabolism in cardiomyopathies (NCT03057002) and in ischemic cardiomyopathies (NCT06047028).

## 7. Current Limitations and Future Perspectives

Overall, hyperpolarized ^13^C imaging presents several advantages over other current noninvasive metabolic imaging techniques. Hyperpolarized scans are fast (<2 min), have no ionizing radiation, and—due to the ability to simultaneously acquire standard magnetic resonance imaging acquisitions—have the potential to directly assess perfusion, ischemia, viability, and altered substrate selection in the same imaging examination. On the other hand, the main limitations of the clinical use of hyperpolarization are complexity and costs, although the hyperpolarization cost is generally lower with respect to the high cost of MR scanners. In particular, specialized equipment in the form of the SPINLab with a sterile fluid pathway and ^13^C cardiac coils is required; during the hyperpolarization process, the fluid path is under severe thermal and mechanical stress, being partly cooled to 1 K superfluid helium and partly heated to 130 °C under a pressure of 16 bar. Moreover, rapid transfer of the sample from the hyperpolarizer to the scanner is still needed to minimize the time between sample dissolution and image acquisition. Differently from positron emission tomography, which is able to detect picomolar amounts of radiolabeled molecules, hyperpolarized imaging requires injection of millimolar concentrations of pyruvate, i.e., a supra-physiological dose, which might theoretically impact the metabolic processes that are being assessed. In perspective, hyperpolarized MR is expected to become cost-effective for specific indications, with similar costs compared to other molecular imaging contrast agents. To preserve low temporal acquisition and processing times and therefore to optimize the experimental design, simulations permitting the evaluation of the influence of SNR on temporal MRS signal analysis were performed and confirmed by in vivo experiments on medium-sized animals injected with hyperpolarized ^13^C-pyruvate [114].

Recent technological advancements (including novel parahydrogen methods, biochemical probes and MR sequences), as well as recent scientific efforts towards standardization of the technology and larger multicenter studies, are paving the way for hyperpolarized ^13^C MR to become much easier to use and more reliable in the near future, with the potential to scale up quickly to more widespread usage [10].

## Figures and Tables

**Table 1:** Gyromagnetic ratios and natural abundance at 3 T [5].

Element	Gyromagnetic Ratios γ (MHz/T)	Natural Abundance (%)
^1^H	42.57	99.9885%
^31^P	11.26	100%
^13^C	10.70	1.07%
^23^Na	17.24	100%
^129^Xe	−11.86	26.44%

**Table 2:** List of most common ^13^C-probes used in pre-clinical and clinical studies.

Metabolite	Pathway	Significance
1-^13^C-pyruvate	Glycolitic pathway	Product of glycolysis, it can be converted to ^13^C-lactate (anerobic conditions) or to acetyl-coA with production of ^13^C-bicarbonate in the mitochondria (oxidation)
^13^C-lactate	Lactate dehydrogenase (LDH)	Derived from ^13^C-pyruvate from LDH (anerobic conditions); increased in cancer cells
^13^C-CO_2_	Pyruvate dehydrogenase (PDH)	Derived as a byproduct of ^13^C-pyruvate conversion to Acetil-CoA
^13^C-bicarbonate	Extracellular pH	Derived from ^13^CO_2_, through extracellular carbonic anhydrase activity
2-^13^C-pyruvate	Tricarboxylic acid cycle (TCA)	The labelled carbon is carried over to acetyl-CoA
^13^C-butyrate	Fatty acid metabolism	
^13^C-acetate	Tricarboxylic acid cycle (TCA) and fatty acid oxidation	Converted to acetyl-CoA by acetyl-CoA synthase
^13^C-alanine	Muscle and liver metabolism	Pyruvate is transaminated to alanine in skeletal muscle; while alanine is deaminated to pyruvate in the liver
^13^C-glucose	pentose phosphate pathway, glycolysis, lactate production	
2-^13^C-dihydroxyacetone	Hepatic gluconeogenesis	
^13^C-glutamine	Mutated isocitrate dehydrogenase (IDH)	In cancer cells, mutated isocitrate dehydrogenase (IDH) converts glutamine to oncometabolite 2-hydroxyglutarate
^13^C-alpha ketoglutarate (αKG)	Mutated isocitrate dehydrogenase (IDH)	In cancer cells, mutated isocitrate dehydrogenase (IDH) converts αKG to oncometabolite 2-hydroxyglutarate and glutamate
^13^C-dehydroascorbate	Redox potential	It is the oxidized form of Vitamin C; it is rapidly converted to [1-^13^C] vitamin C within the liver, kidneys, brain and tumors
^13^C-acetoacetate	Mitochondrial redox status	
^13^C-glutathione	Antioxidant and redox status	Antioxidant synthesized from glutamate (glu), cysteine (cys) and glycine (gly)
^13^C-cystine	Antioxidant and redox status	Component of glutathione
^13^C-urea	Perfusion	Inert metabolic probe
^13^C-fumarate	Necrosis	In case of cell death, exogenous ^13^C-fumarate is converted to ^13^C-malate by intracellular fumarase (released in the extracellular space)
^13^C-malate	Necrosis	Absent in healthy cells, while produced from ^13^C-fumarate by extracellular fumarase released by necrotic cells

**Table 3:** Pre-clinical cardiovascular studies in large animal models.

Author	Animals	Scanner	Spatial Resolution	Sequence	Post-Processing	Disease	Results
Agger et al., 2020 [110]	5 pigs	3 T HDx (GE Healthcare, Waukesha, WI, USA)	1.01 × 1.45 mm^2^	Cardiac triggered 2D ^13^C IDEAL spiral	Not reported	Pulmonary banding	Increase in the lactate/bicarbonate ratio compared with healthy control
Schroeder et al., 2013 [78]	5 pigs	3 T MR750 (GE Healthcare, Waukesha, WI, USA)	9 mm		SAGE™ software (GE Healthcare) MATLAB (MathWorks, Natick, MA, USA)	Dilated cardiomyopathy	Reduced pyruvate oxidation
Golman et al., 2008 [83]	10 pigs (5 with 15 min occlusion, 5 with 45 min occlusion)	1.5T Magnetom Sonata (Siemens Medical Solutions, Erlangen, Germany)	7.5 mm	^13^C CSI	in house developed software	Effect of coronary artery occlusion	15-min occlusion: bicarbonate reduces in diseased area; 45-min occlusion: ^13^C-bicarbonate and ^13^C-alanine signal reduced in the diseased area
Lewis et al., 2018 [111]	7 pigs	3 T MR750 3 T MR750 (GE Healthcare, Waukesha, WI, USA)	10.7 mm	Spiral sequence	Not reported	Myocardial infarction after coronary artery balloon-occlusion	Increase ^13^C-lactate signal in infarct. No significant difference in ^13^C-bicarbonate signal
Aquaro et al., 2015 [113]	7 pigs	3 T HDx TWINSPEE 3 T MR750 (GE Healthcare, Waukesha, WI, USA)	15 mm	3D-IDEAL spiral CSI	MATLAB (MathWorks, Natick, MA, USA)	Ischemic myocardium after pneumatic occlusion	Increase ^13^C-lactate signal; reduced ^13^C-bicarbonate within the area at risk
Fuetterer et al., 2022 [14]	8 pigs	3 T (Philips Medical Systems, Best, The Netherlands)	1 mm	Customized spatial-spectral excitation (IDEAL approach)	MRecon (GyroTools LLC, Zurich, Swizerland)	Catheter-based 90-min occlusion	Elevated lactate-to-bicarbonate ratios at day 6 after infarction
Chen et al., 2012 [77]	Not reported	3 T MR750 (GE Healthcare, Waukesha, WI, USA)	Not reported	Pulse-acquire sequence	SAGE™ software (GE Healthcare)	Healthy pig	Feasibility of using dual-labeled hyperpolarized [1,2-^13^C_2_]pyruvate as a substrate for dynamic cardiac metabolic MRS studies
Fuetterer et al., 2016 [67]	6 pigs	3 T Ingenia wide-bore scanner (Philips, Best, The Netherlands)	3 mm	Velocity-selective binomial excitation scheme	MRecon (GyroTools LLC, Zurich, Switzerland)	Healthy pig	Potential of hyperpolarized ^13^C-urea imaging for diagnostic purposes.

**Table 4:** Human cardiovascular studies.

Author	Subjects	Scanner	Spatial Resolution	Sequence	Post-Processing	Disease	Results
Cunningham et al., 2016 [74]	4	3 T MR750 (GE Healthcare, Waukesha, WI, USA)	8.8 mm	Slice-selective spectral-spatial excitation	Not reported	Healthy subjects	^13^C-bicarbonate in this healthy cohort
Apps et al., 2021 [12]	2	3 T Tim Trio (Siemens Medical Solutions, Erlangen, Germany)	Not reported	Hybrid-shot spiral	AMARES algorithm	Myocardial Infarction	Reduced PDH-mediated aerobic conversion to ^13^C-bicarbonate
Rider et al., 2020 [15]	13 (Diabetes) 12 (healthy group)	3 T Tim Trio (Siemens Medical Solutions, Erlangen, Germany)	8 mm	Pulse-acquire spectroscopy	Not reported	Diabetes mellitus	^13^C-bicarbonate reduced
Joergensen et al., 2022 [76]	6	Not reported	13.3 mm	Spectral-spatial (SPSP) excitation with spiral read-out	MATLAB (MathWorks, Natick, MA, USA)	Healthy subjects	Increased pyruvate oxidation during low to moderate cardiac stress
Chen et al., 2024 [75]	3	3 T MR750 (GE Healthcare, Waukesha, WI, USA)	Not reported	Dynamic slab spectroscopy	MATLAB (MathWorks, Natick, MA, USA)	Healthy subjects	Cardiac metabolite measurement in the fasting/fed states provides information on cardiac metabolic flexibility and the acetylcarnitine pool.

## Data Availability

Not applicable.
